# The Central Role of Redox-Regulated Switch Proteins in Bacteria

**DOI:** 10.3389/fmolb.2021.706039

**Published:** 2021-07-02

**Authors:** Rosi Fassler, Lisa Zuily, Nora Lahrach, Marianne Ilbert, Dana Reichmann

**Affiliations:** ^1^ Department of Biological Chemistry, The Alexander Silberman Institute of Life Sciences, Safra Campus Givat Ram, The Hebrew University of Jerusalem, Jerusalem, Israel; ^2^ Aix-Marseille University, CNRS, BIP, UMR 7281, IMM, Marseille, France

**Keywords:** thiol-switches, oxidative stress, redox-regulated proteins, Hsp33, metal induced oxidation, oxidative stress in prokaryotes

## Abstract

Bacteria possess the ability to adapt to changing environments. To enable this, cells use reversible post-translational modifications on key proteins to modulate their behavior, metabolism, defense mechanisms and adaptation of bacteria to stress. In this review, we focus on bacterial protein switches that are activated during exposure to oxidative stress. Such protein switches are triggered by either exogenous reactive oxygen species (ROS) or endogenous ROS generated as by-products of the aerobic lifestyle. Both thiol switches and metal centers have been shown to be the primary targets of ROS. Cells take advantage of such reactivity to use these reactive sites as redox sensors to detect and combat oxidative stress conditions. This in turn may induce expression of genes involved in antioxidant strategies and thus protect the proteome against stress conditions. We further describe the well-characterized mechanism of selected proteins that are regulated by redox switches. We highlight the diversity of mechanisms and functions (as well as common features) across different switches, while also presenting integrative methodologies used in discovering new members of this family. Finally, we point to future challenges in this field, both in uncovering new types of switches, as well as defining novel additional functions.

## Introduction

Most bacterial cells live in a dynamically fluctuating environment, requiring rapid responses to enable successful growth. These changing environments might induce stress conditions (e.g., oxidative stress, heat shock, etc.), which challenge bacterial homeostasis and macromolecules, affecting a wide variety of cellular processes. Thus, it is not surprising that bacteria and other organisms evolved different sensors and first line of defense mechanisms to combat environmental assaults. One such stress-response strategy utilizes rapid post-translational modifications of proteins that induce the general response and trigger defense activities. Reversible post-translational modification of proteins is one of the major toolboxes available to cells, which ensures a plasticity of the cellular proteome, as well as rapid control of diverse cellular functions, including stress specificity. While phosphorylation is one of the major regulators of the cell cycle (Garcia-Garcia et al., 2016), oxidation, protonation, and chlorination were found to be crucial to alter the activity of specific proteins during oxidative and acidic conditions (Winter et al., 2008; Palumaa, 2009). There are multiple benefits of post-translational switches: rapid reactivity, tight control, reversibility and low energetic cost relative to transcription and translation of new proteins (Venne et al., 2014; Vu et al., 2018; Macek et al., 2019).

Prokaryotes constantly produce reactive oxygen species, ROS, (peroxide, superoxide and others) during their life cycle as a consequence of growth in an aerobic environment (Zhao and Drlica, 2014; Van Loi et al., 2015). These ROS can be byproducts of either oxidoreductase reactions or oxidation of univalent electron donors such as metal centers, sulfur and others. In addition to the self-produced oxidants, bacteria is exposed to environmental ROS originating from (i) oxidative bursts of phagocytic cells during the host immune defense (Hardbower et al., 2013); (ii) irradiation of water; (iii) oxidation of pollution chemicals found in the bacterial growth environment; (iv) oxidant excretion by other bacterial and eukaryotic species into the common habitat environment (Imlay, 2019; Reniere, 2018).

Abnormal levels of oxidative stress can cause irreversible damage to diverse cellular macromolecules including nucleotides, lipids and proteins, affecting their function and stability. The exposure of bacteria to harmful oxidation has led to the evolution of extensive damage repair systems which consist of a large repertoire of antioxidant enzymes that detoxify different oxidants and convert them into harmless molecules (Ezraty et al., 2017). The main players of the damage repair system include peroxiredoxins [AhpC, (Perkins et al., 2015)], catalases (Yuan et al., 2021) and superoxide dismutases [SOD, (De Groote et al., 1997)] which detoxify peroxide and superoxides, as well as glutaredoxins [gpxA, (Moore and Sparling, 1995)] and thioredoxins, which restore protein thiols in cellular proteomes. Many wonderful reviews were written about the damage repair system in bacteria and eukaryotes, among which are (Verity, 1994; Visick and Clarke, 1995; Cabiscol et al., 2000; Mitra et al., 2002; Hanschmann et al., 2013; Ezraty et al., 2017).

Non-specific protein oxidation might lead to various post-translational modifications of sulfur-containing residues (Cys and Met) and aromatic residues (Tyr, Trp), as well as induce undesirable disulfide bonds and affect protein cofactors, especially metal centers – all which might lead to protein inactivation, unfolding, accumulation of toxic aggregates and even cell death (Ilbert et al., 2006; Chung et al., 2013; Dahl et al., 2015; Kehm et al., 2021).

This is alongside a beneficial role of intracellular oxidants in biosynthesis, lipid oxidation, metabolism and environmental response (Brynildsen et al., 2013; Imlay, 2013; McBee et al., 2017), which requires development of a highly sensitive and dynamic mechanism to maintain the balance between oxidation and cellular homeostasis. Elegant studies by Imlay and Linn (1986) and (Rodríguez-Rojas et al., 2020) showed that priming *Escherichia coli* with low levels of peroxide increases its survival during severe oxidative stress conditions, emphasizing the importance of dynamic responses and bacterial adaptation to changing intracellular ROS levels. Moreover, it was shown that production of intracellular ROS might provide antibiotic tolerance in Mycobacteria, suggesting a tight regulation between redox homeostasis and adaptation pathways (McBee et al., 2017).

Therefore, prokaryotes have developed multi-level approaches to sense changes in redox homeostasis (by SoxR, OxyR, and RsrA), to detoxify undesirable levels of ROS (through scavenging enzymes such as catalase, superoxide permutate, peroxidase) and to protect the cellular proteome against potential damage (by Hsp33 chaperone, thioredoxin, and others).

One of the main strategies of this defense system is to utilize rapid and reversible oxidation-dependent modification of specific protein thiol residues, serving as redox-sensitive switches of the defense proteins and mediating their rapid activation (Ilbert et al., 2006; Cremers and Jakob, 2013).

Another strategy – which can be coupled to modification of the thiol groups – is exploiting redox properties of metal centers to regulate proteins during fluctuating oxidant levels. Thus, bacteria (and eukaryotes) have developed an elegant way to convert “protein weakness” into a powerful and robust mechanism to regulate the expression of genes that provide a defense against oxidative stress. They are then able to detoxify ROS using reversible reduction-oxidation cycles of catalytic cysteine residues or cofactors, restore the redox status of proteins and maintain protein quality control under stress conditions. Here, we will briefly discuss different types of protein switches and their working mechanism, which enable bacteria to adapt and defeat oxidation-related challenges during their life cycle.

### Protein Thiols – The Central Component of Antioxidant Protein Switches

**FIGURE 1:**
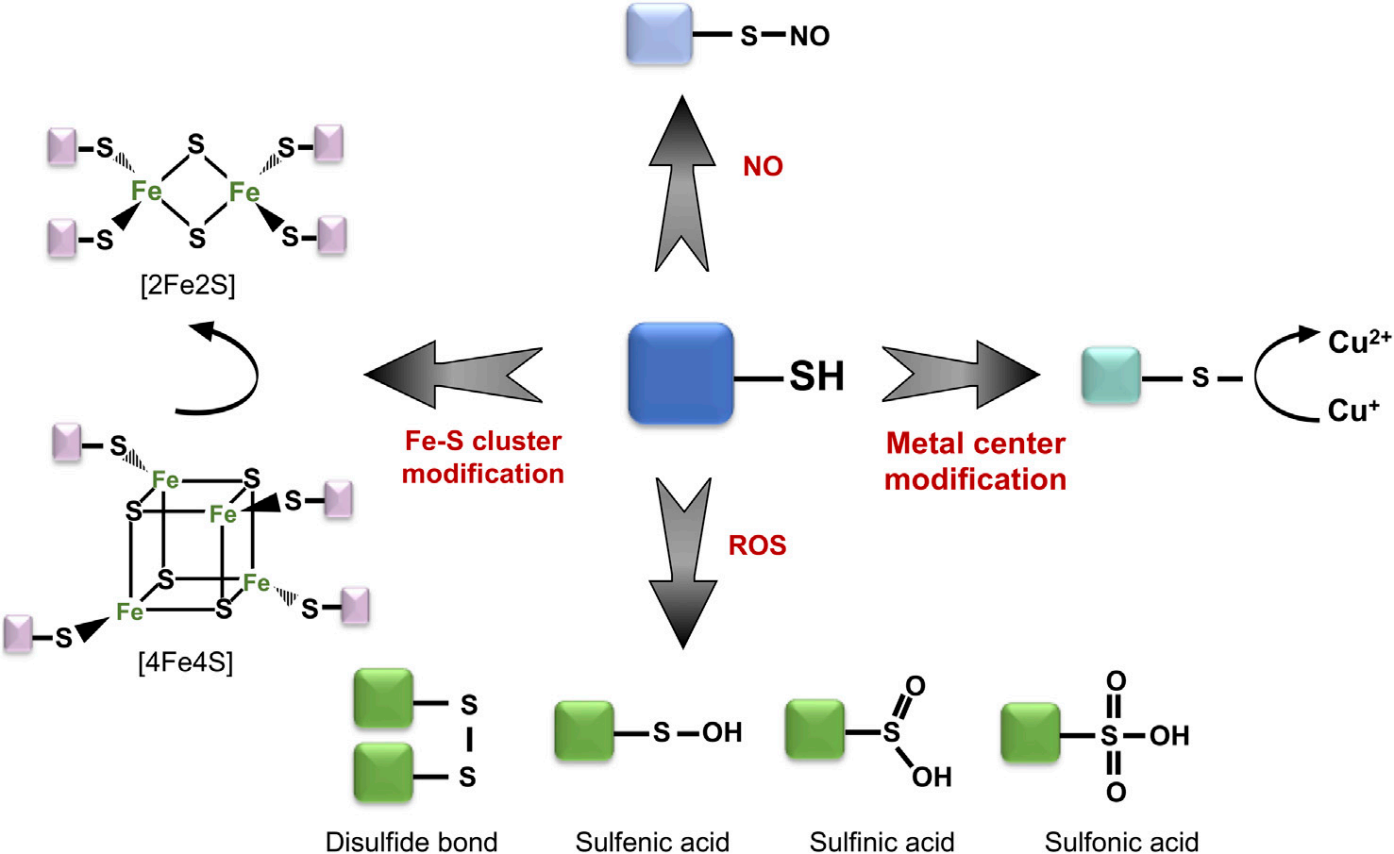
Thiol group might serve as a functional switch. Reactive protein thiols can undergo a wide range of modifications depending on the oxidative stress conditions. These include both reversible (e.g., sulfenic acid, disulfide bridge formation) and irreversible modifications (e.g., sulfinic acid, sulfonic acid). Moreover, thiol groups can interact with metal centers and play central roles in the detection of redox change.

The aerobic lifestyle is an inevitable source of intracellular ROS, producing byproducts such as hydrogen peroxide (H_2_O_2_), hydroxyl (·OH) and superoxide anion (O^−2^) radicals. Accumulation of these ROS results in negatively charged modification of reactive protein thiols, in the form of sulfenic (-RSOH) or sulfinic acids (-RSO_2_H), or in the formation of non-native, covalent disulfide bonds within and between different proteins (Figure 1) (Georgiou, 2002; Ilbert et al., 2006).

**FIGURE 2:**
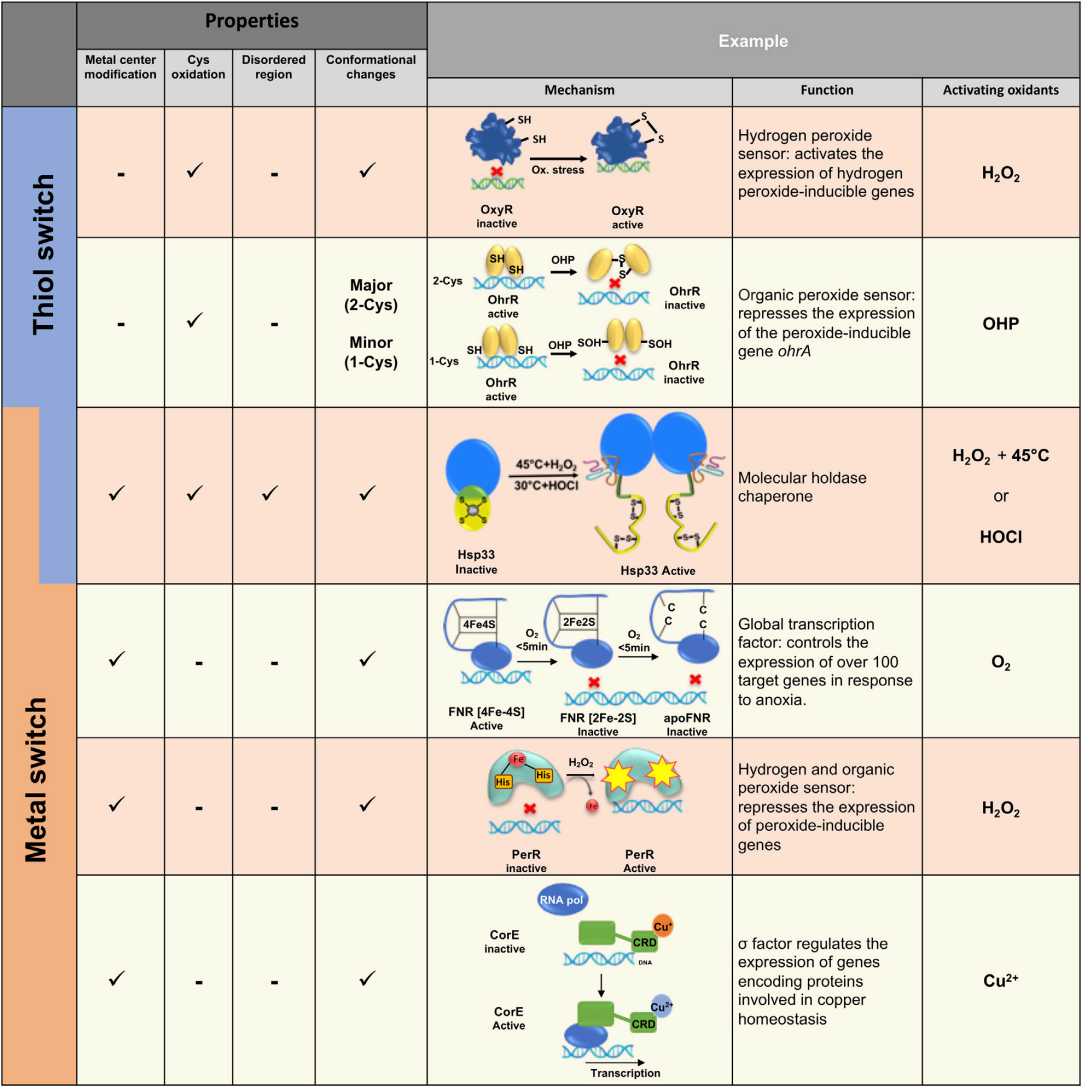
Examples of various thiol and/or metal switches in bacteria. Different thiol and metal center switches regulate redox homeostasis of bacteria at different levels, ranging from gene expression to anti-aggregation activity. Different mechanisms of redox-regulation activity are presented.

Such thiol oxidation of cysteine and methionine residues might induce local structural and chemical alterations, influence binding of metal centers, as well as form new, non-native protein complexes, conjugated *via* disulfide bonds. While a majority of proteins undergo a loss of function or misfolding upon oxidation, cells have developed an array of different thiol-switch proteins, which utilize site-specific oxidation for their activity. The majority of known thiol switch proteins contain reactive cysteine residues which can “sense” changes in the redox status of cells and undergo reversible modifications, which regulate their activation or inactivation (Figure 2) (Antelmann and Helmann, 2011; Fra et al., 2017). Reactive thiols of these thiol-switch proteins usually have unique chemical properties, while some of the thiols themselves are located in structurally flexible and conserved regions. These thiols can be modified in various ways: sulfenylation, nitrosylation, chlorination, glutathionylation, persulfidation, and disulfide formation, responding to different oxidants (Figure 1). Reduction of these modifications is done by specific enzymes which restore the redox status of thiols (e.g., thioredoxins or glutaredoxin) and by related cofactors such as glutathione (GSH) and its analogs [e.g., mycothiol (MSH) in Actinobacteria, bacilithiol (BSH) in Firmicutes] (Fahey, 2013), as well as NAD(P)H (Reniere, 2018).

Despite the wide diversity of bacterial antioxidant strategies, the most studied thiol-switch proteins are ones that use highly reactive cysteine thiolates as a switch. This is most probably due to the availability of a diverse range of experimental tools, ranging from thiol trapping, thiol quantification and redox mass spectrometry that allows investigation of the redox status of cysteine thiols.

One of the classic examples of a thiol switch protein in bacteria is a transcriptional factor OxyR, which was first identified in *E. coli* (Christman et al., 1989) and *S. typhimurim* (Christman et al., 1985; Morgan et al., 1986). Stamler, Storz and others showed that hydrogen peroxide and S-nitrosothiols activate OxyR transcriptional activity, leading to the production of ∼130 proteins with antioxidant and anti-nitrosylation activities (Seth et al., 2020). OxyR activity is induced by oxidation of a highly conserved Cys residue (Cys199 in *E. coli*), which undergoes sulfenylation (S-OH) and consequent disulfide formation with the adjacent cysteine (Cys 208 in *E. coli*) (Zheng et al., 1998). Disulfide bond formation induces major structural rearrangement by forming a new beta strand in the protein, altering OxyR’s binding to its promoter and subsequent recruitment of RNA polymerase (Figure 2) (Fuangthong and Helmann, 2002; Georgiou, 2002) Interestingly, S-nitrosylation of Cys199 leads to an alternate response to combat nitrosative stress rather than oxidative stress conditions, by inducing the expression of enzymes which detoxify NO species involved in SNO metabolism during aerobic and anaerobic conditions.

This ultimately results in different DNA binding affinity and specificity, (Kim et al., 2002; Seth et al., 2020), depending on the respective stress conditions. This makes OxyR a notable, multi-sensing thiol-switch protein, which uses stress-specific structural plasticity to activate differential response pathways to overcome oxidative or nitrosative stress. It is reasonable to speculate that other thiol modifications of Cys199 might lead to activation of other related stress-response pathways.

Another example of a thiol-switch sensor is the very well-studied family of OhrA peroxidase repressors, named OhrR, which bind to the OhrA promoter in the reduced form (Figure 2). OhrR repressors are part of the MarR-family regulators, protecting bacteria against a wide range of oxidants, reactive nitric species and reactive electrophilic species (Hillion and Antelmann, 2015). Specifically, OhrR is activated by organic hydroperoxides (OHP) and other ROS (Van Loi et al., 2015; Sun et al., 2018; Ruhland and Reniere, 2019). The OhrR repressor family can be divided into two classes, 1-Cys (first identified in *Bacillus subtilis* (Fuangthong et al., 2001) and 2-Cys [first identified in *Xanthomonas campestris* (Sukchawalit et al., 2001)]. These harbor either one or two redox-sensitive thiol groups in the N-terminal region, adjacent to the DNA binding domain. Despite high sequence and functional similarity, these two classes represent different peroxide-sensing mechanisms.

The *B. subtilis* OhrR 1-cys repressor is inactivated by organic peroxide *via* formation of sulfenate (-RSOH) on Cys 15 and small local structural changes (Hong et al., 2005; Duarte and Latour, 2010), which lead to a rapid reaction with low molecular weight (LMW) thiols and the formation of reversible mixed disulfides, including S-BSH (Lee et al., 2007). Moreover, non-reversible, overoxidation of Cys15 to sulfinic (-RSO_2_H) or sulfonic (-RSO_3_) acid leads to the detachment of OhrR from the *ohrA* operator region (Soonsanga et al., 2008b).

In contrast to the OhrR 1-cys repressor, *X. campestris* OhrR is inactivated when the initially oxidized Cys22 reacts with another conserved Cys127, located >15Å apart, in the C-terminal domain of the opposing side of the dimer (Newberry et al., 2007). This inter-subunit disulfide bond induces a massive conformational change and rotation of the oxidized OhrR dimer, resulting in dissociation from the operator region and expression of the OhrA peroxidase. Conditional disulfide bond formation is a more robust mechanism than oxidation of a single thiol since it does not rely on the presence of LMW thiols and thus prevents irreversible oxidation of the active thiol groups of the regulatory protein (Soonsanga et al., 2008a).

The 1-Cys and 2-Cys transcriptional factors are common in bacteria and eukaryotes. These mechanisms are utilized in order to sense a diverse repertoire of stresses which challenge redox homeostasis and a functional proteome. The detailed mechanisms and regulation of such thiol-switch regulators in bacteria are wonderfully described by Antelmann and Helmann (2011), Jakob and Reichmann (2013), Hillion and Antelmann (2015), Vázquez-Torres (2012), Boronat et al., (2014) and many others.

One of the main classes of thiol switches in bacteria (and eukaryotes) are thioredoxin and glutaredoxin enzymes that restore the redox status of proteins using reduction-oxidation cycles of their conserved catalytic cysteine residues with the help of cellular cofactors, such as NADH, NADPH, and Glutathione (Holmgren et al., 2005; López-Grueso et al., 2019). Numerous fantastic reviews have been written about the detoxification properties of thioredoxin and glutaredoxin in bacteria and the role of their reversible thiol modifications in maintaining redox homeostasis (Zeller and Klug, 2006; Berndt et al., 2008; Jacquot and Zaffagnini, 2019).

### Redox Regulation by Using Metal Centers

Metalloproteins are central actors in a wide number of biological processes (Waldron and Robinson, 2009). The chemistry of metals brings unique properties to enzymes, allowing the catalysis of redox reactions required for essential pathways such as respiration, nitrogen fixation, water oxidation and others (Liu et al., 2014). Redox-mediated metalloproteins usually contain transition metals (e.g., Fe, Zn, and Cu) that have multiple oxidation states by their nature and can therefore sense and regulate protein function. Such metallocenters can mediate various pathways in cells by using different redox-regulating mechanisms: (i) by changing the redox state of the metal center, (ii) by modification of the metallocenter composition, or (iii) by the loss of the metal center after its oxidation. Such modifications trigger the activation/inactivation of the metalloproteins either by conformational changes or by altering protein-protein interactions.

While many metalloproteins and their cofactors are sensitive to oxidation and might even release ROS *via* a Fenton reaction [e.g., iron-sulfur (Fe-S) clusters], (Imlay, 2006), other proteins use this sensitivity as a redox switch for their activity. For instance, the oxygen sensor FNR (fumarate nitrate reductase regulator) regulates the expression of hundreds of genes involved in anaerobic metabolism (Kang et al., 2005; Kiley and Beinert, 1998; Mettert and Kiley, 2018) (Figure 2). In the absence of oxygen, the active form of FNR is a DNA-binding homodimer, containing one [4Fe-4S] cluster per monomer, bound to four highly conserved cysteine residues (Lazazzera et al., 1996). In aerobiosis conditions, the FNR [4Fe-4S] cluster rapidly decays into [2Fe-2S] with a release of two S and Fe^3+^ ions as well as a superoxide ion (O_2_^−^), which is further converted to peroxide and water (Crack et al., 2007). Longer oxidation generates an inactive, monomeric apo-FNR form, lacking the [2Fe-2S] cluster (Lazazzera et al., 1996; Khoroshilova et al., 1997; Reinhart et al., 2008). Similar to OxyR, FNR has a dual role in the regulation of genes responding to either oxidative or nitrosylation stress, by differential modification of the switch centers. Upon increased levels of nitric oxide (NO), the metal center is converted into an Fe-NO_4_ cluster accompanying oxidation of the catalytical cysteines in the metal cluster, resulting in monomerization of FNR (Crack et al., 2013). Despite decades of FMN research, only recently the X-ray structure of FNR from *Aliivibrio fischeri* was resolved. This provided insights into the catalytic mechanism of the [4Fe-4S] - [2Fe-2S] exchange mediating FNR monomerization, (Volbeda et al., 2015), with the structural analysis uncovering a cascade of structural rearrangements induced by oxidation of the metal cluster. This indirectly leads to a breakage of salt bridges as well as of the helical interface which maintains the dimer conformation of inactive FNR during reducing conditions (Volbeda et al., 2015; Mettert and Kiley, 2018).

Metal’s oxidation might lead to modification of residues found in the metal’s vicinity. PerR from *B. subtilis* is an excellent example of a protein utilizing this reactivity for functional activation (Pinochet-Barros and Helmann, 2018). PerR is a repressor known to belong to the Fur family of proteins (ferric-uptake repressor). Under physiological growth conditions, PerR is a dimer containing two metal-binding sites, Zn^2+^ and Fe^2+^ per monomer (Traore et al., 2006; Ma et al., 2011). Upon exposure to low concentrations of H_2_O_2_, PerR induces expression of genes involved in the detoxification of peroxide and related damage (Helmann et al., 2003). Through a mechanism called metal-catalyzed oxidation (MCO), H_2_O_2_ reacts with the bound Fe^2+^, leading to the oxidation of one of the two histidines involved in coordinating with the iron atoms (Lee and Helmann, 2006). Histidine oxidation induces PerR conformational changes, which triggers its release from DNA (Ahn and Baker, 2016) (Figure 2). In contrast to other peroxide-sensing transcription factors described above, PerR’s regulatory mechanism is not cysteine but histidine-dependent. Interestingly, the Fe^2+^ binding site can also bind Mn^2+^, depending on the relative amount of both metals in the growth media. At a high concentration of Mn^2+^, the PerR regulon is tightly repressed even in the presence of peroxide, highlighting the importance of the MCO mechanism and its high dependence on the presence of iron in the media (Fuangthong et al., 2002).

Metal centers in biological systems can oscillate between a reduced and oxidized form, where such redox changes frequently allow electron transfer to occur. In some cases, however, these redox status modifications change the protein function and may be considered as an additional redox-regulation mechanism. The regulator SoxR, for example, has been well-described to stimulate the transcription of SoxS exclusively in presence of redox-cycling compounds (Gaudu and Weiss, 1996; Imlay, 2015; Outten and Theil, 2009). SoxR is a dimer where each monomer contains a [2Fe-2S] cluster (Hidalgo et al., 1995). In its reduced form [2Fe-2S]^+^, SoxR binds DNA without inducing SoxS transcription, whereas in its oxidized form [2Fe-2S] ^2+^, SoxR induces SoxS expression (Ding et al.,1996; Gaudu et al., 1997) (Figure 2). Oxidation of SoxR leads to slight conformational changes, resulting in a distortion of the bound DNA and modify RNA polymerase transcription (Kobayashi et al., 2011).

Another recently discovered protein, a copper-binding regulator CorE from *Myxococcus xanthus*, is also regulated *via* a redox mechanism (Gómez-Santos et al., 2011; Muñoz-Dorado et al., 2012). Indeed, oxidized copper (Cu^2+^)-CorE binds to DNA whereas Cu^+^-bound CorE does not (Gómez-Santos et al., 2011) (Figure 2).

### Hsp33 – An Example for Utilizing Redox-Regulated Protein Plasticity to Maintain Proteome Functionality During Oxidative Stress Conditions

Around 20 years ago, the Hsp33 chaperone was discovered as a first line of defense chaperone protecting bacterial proteins against aggregation during oxidative stress in *E. coli* (Hoffmann et al., 2004). Since then, additional homologues of Hsp33 were identified and characterized in other bacterial species as well as in unicellular algae (Segal and Shapira, 2015) and pathogens (Trypanosoma and Leishmania) (Aramin et al., 2020). This highlights Hsp33 as a promising new drug target against bacterial and Trypanosoma pathogens.

Hsp33 is one of the crucial ATP-independent holdases (or holding chaperones), which is activated under conditions that lead to protein misfolding and accumulation of toxic aggregates, such as oxidative unfolding. Hsp33 “senses” the presence of oxidants or chlorine species (e.g., HOCl) through a highly reactive Zn center, comprising of four completely conserved cysteines forming CXCX and CXXC motifs harboring one Zn^2+^ ion in the inactive, reduced form (Ilbert et al., 2006; Aramin et al., 2020). Oxidation triggers Zn release, formation of two disulfide bonds and rapid unfolding of almost half of the protein, which exposes hydrophobic regions involved in the anti-aggregation activity of Hsp33 (Rimon et al., 2017). Oxidation per se of the Zn center is not sufficient for converting Hsp33 into a potent holdase, and requires additional unfolding conditions (e.g., mild heat or the acidity of HOCl). Upon return to normal conditions, reduction of the Zn center leads to refolding of Hsp33 (Ilbert et al., 2007; Cremers et al., 2010; Rimon et al., 2017), destabilization of the bound client protein and transfer to the foldase chaperone system, DnaK/J (Reichmann et al., 2012a) (Figure 2). This working cycle of Hsp33 provides a unique mechanism of a thiol switch protein which uses a redox-dependent metal center and a disorder-to-order transition for its function. Thus, the Hsp33 protein family preserves not only reversible catalytical centers, but reversible structural plasticity underlying the Hsp33 function as well.

### Integrative Methodology Assists in Identifying Redox Switch Proteins and Future Directions

Technological progress over the last few years has drastically advanced the discovery of new redox switches and allowed the community to deepen the understanding of the complex redox-regulating mechanisms that are vital in defining the fate of bacteria. Due to their diversity and elusive nature, research of redox switches requires a multidisciplinary toolbox of techniques combining biochemistry, redox chemistry, structural and cell biology.

The majority of technological efforts and breakthroughs have been invested in uncovering thiol-redox switches and the related pathways. This is due to the importance and high conservation of cysteines, which usually have a crucial role in protein structure and function. Moreover, redox chemistry has provided existing tools to investigate thiol-redox reactions that could be adopted to biological systems. Therefore, it is not surprising that many of the redox-switch proteins that were discovered in recent years are thiol-switch proteins. These have been studied by different approaches, ranging from single cysteine substitution (usually to serine), *in vivo* and *in vitro* thiol trapping analyses, to system-wide redox proteomics (Rudyk and Eaton, 2014; Allan et al., 2016; Botello-Morte et al., 2016; van der Reest et al., 2018).

During the last decade, several studies showed that antibiotic treatment alters redox homeostasis and leads to the accumulation of ROS in bacteria, which might be an additional cause for cell death (Kohanski et al., 2007; Van Acker et al., 2016). While the mechanism is not clear, recent studies took an advantage of the ROS-antibiotics relationship to utilize ROS as an antibacterial treatment. For example, Antelman’s lab showed that antimicrobial treatment by AGXX results in ROS intracellular production, which targets multi-drug resistant pathogens (Van Loi et al., 2018; Linzner et al., 2021). This study raises many questions regarding the potential role of indirect ROS accumulation and associated thiol-switch proteins in cells challenged by antibiotics and the multi-drug resistance processes. This intriguing correlation should be further investigated.

To date, redox biologists have an array of innovative tools to differentially label reduced and oxidized cysteine residues *in vivo* or *in vitro*, in order to detect changes in the redox status of either single or multiple proteins in a biological sample (Rudyk and Eaton, 2014). This includes a collection of diverse alkylating reagents (e.g., maleimide, iodoacetate, and their derivatives) which specifically react with the thiol groups of cysteine residues, which can then be used to quantify the total change in reduced thiols in cells, detect changes in a specific cysteine thiol of protein of interest, or in the entire proteome (Rudyk and Eaton, 2014; Winther and Thorpe, 2014; Alcock et al., 2018). Conjugation of alkylating reagents with biotin molecules has opened up a new opportunity to investigate the interactomes of potential thiol-switch proteins *in vivo* and to define the redox-dependent dynamics of these interactions. A combination of genetics, thiol trapping, and structural biology approaches have enabled definition of the redox-dependent mechanism of essential thiol switches, such as Hsp33, OxyR, and many others, providing a deeper knowledge on both the protein and system levels (Choi et al., 2001; Ilbert et al., 2007).

One of the breakthroughs in redox biology was the development of redox proteomics workflows (Zaccarin et al., 2014; Gu and Robinson, 2016; Duan et al., 2017). Since ∼10% of residues are cysteines, one of the main challenges in redox proteomics is the ability to capture and isolate thiol proteomes while minimizing non-specific oxidation induced during sample preparation and by the mass spectrometer itself. Leichert and Jakob, among others, have established a highly efficient proteomic workflow, named OxICAT. This workflow uses differential labeling by biotinylated, isotope-coded light and heavy affinity tags with an iodoacetamide reactive group (ICAT). Quantification of the redox profile of cysteines is based on a ratio approach, which allows for overcoming potential artifacts that follow protein abundance, as well as proteins lost during the sample preparation steps. The OxICAT method has not only uncovered novel, potential thiol switch proteins across the proteome, but remarkably has established a mechanistic link between reversible oxidation and aging in eukaryotic cells, pointing toward pathways and kinetics of thiol oxidation during age or following different growth conditions. In bacteria, OxICAT and other redox proteomics techniques identified redox-regulated metabolic pathways associated with phagocytosis (Leichert et al., 2008), as well as a bacterial redox-regulated response toward antibacterial treatment (Reiter et al., 2020). Moreover, the same platform was adopted to uncover a cross-reactivity of cysteine thiols to different oxidants and modifications, such as nitrosylation (Leichert and Jakob, 2006), chlorination (Chen et al., 2016), mycothiolation (Hillion et al., 2017), and sulfhydration (Zivanovic et al., 2019), defining the plasticity and versatility of the thiol-switch proteins in bacteria.

While redox proteomics can point to a potential key redox player, a detailed biochemical and biophysical analysis should be done to investigate the reaction mechanism. As previously mentioned, high-resolution structural methods [e.g., NMR (nuclear magnetic resonance) and X-Ray crystallography] were able to define the exact redox cascade mechanism in metalloproteins and define catalysis at the atomic level. This is challenging in the case of redox switch proteins, which require structural plasticity or oligomerization (e.g., Hsp33) for their activity, which complicate the obtention of an atomic structure using NMR or X-Ray. In this case, structural mass spectrometry (native MS and Hydrogen-deuterium-exchange, HDX-MS) takes on its undebated role. HDX-MS analysis of Hsp33’s working cycle has enabled mapping of redox-dependent conformational changes on both the chaperone and its substrate, mediating substrate binding and release (Reichmann, 2012b; Fassler et al., 2018).

Furthermore, research on redox-regulating metalloproteins sits in the junction between structural biology and chemistry. During the last decade, biophysical approaches such as UV-Visible, EPR (electron paramagnetic resonance), NMR or X-ray absorption spectroscopy have pushed the metal-switch field forward, providing high resolution mechanisms of enzymes and transcriptional factors. However, to date, metal-switch proteins have been mainly described *in vitro* on purified systems. The recent development of in cell-NMR and in cell-EPR will give a better picture of *in vivo* metal-switch reactions. In addition, finding new family members might be possible with the development of metallomics approaches combined with spectroscopy or other tools, to find redox-regulated metal centers.

However, despite the many fascinating breakthroughs that have been made over the past several years, we need to develop a new repertoire of methodologies addressing non-thiol regulation. Recently, a few technologies were established to investigate methionine and tyrosine oxidation, however, more should be done in this field. The development of these methodologies will open a door to uncover new types of switch proteins, employing other regulatory sites and chemistry.

Another aspect that should be addressed in the redox-switch protein research is the multi-functionality of this class of proteins. It is clear that many redox-regulated proteins cannot be simply defined by loss-gain of function under oxidation-reduction conditions. Many of the redox-regulated proteins have more than one biological function and specificity to different radicals. One of the next challenges in the field is to understand the evolutionary path of redox switch proteins, their multiple functionality, and their reactivity.
